# Simulation and social network analysis provide insight into the acquisition of tool behaviour in hybrid macaques

**DOI:** 10.1098/rspb.2022.2276

**Published:** 2023-03-29

**Authors:** Jonathan S. Reeves, Amanda Tan, Suchinda Malaivijitnond, Lydia V. Luncz

**Affiliations:** ^1^ Technological Primates Research Group, Max Planck Institute for Evolutionary Anthropology, Deutscher Platz 6, 04103 Leipzig, Germany; ^2^ Department of Anthropology, Durham University, Durham DH1 3LE, UK; ^3^ Department of Biology, Faculty of Science, Chulalongkorn University, Bangkok 10330, Thailand; ^4^ National Primate Research Centre of Thailand, Chulalongkorn University, Saraburi 18110, Thailand

**Keywords:** tool use, skill acquisition, agent-based modelling, social network analysis

## Abstract

The pathways through which primates acquire skills are a central focus of cultural evolution studies. The roles of social and genetic inheritance processes in skill acquisition are often confounded by environmental factors. Hybrid macaques from Koram Island (Thailand) provide an opportunity to examine the roles of inheritance and social learning to skill acquisition within a single ecological setting. These hybrids are a cross between tool-using Burmese long-tailed (*Macaca fascicularis aurea*) and non-tool-using common long-tailed macaques (*Macaca fascicularis fascicularis*). This population provides an opportunity to explore the roles of social learning and inheritance processes while being able to exclude underlying ecological factors. Here, we investigate the roles of social learning and inheritance in tool use prevalence within this population using social network analysis and simulation. Agent-based modelling (ABM) is used to generate expectations for how social/asocial learning and inheritance structure the patterning in a social network. The results of the simulation show that various transmission mechanisms can be differentiated based on associations between individuals in a social network. The results provide an investigative framework for discussing tool use transmission pathways in the Koram social network. By combining ABM, network analysis, and behavioural data from the field we can investigate the roles social learning and inheritance play in tool acquisition of wild primates.

## 1. Introduction

The emergence of tool use is argued to have facilitated the adaptive success of the human lineage [1]. Thus, the pathways through which tool use is transmitted across generations are a primary focus of cultural evolution studies [2–4]. While there is a consensus that tool use arises as the result of a combination of genetic and social processes, the contribution of these factors and their evolutionary implications continues to be debated [5–11]. According to the cognitive niche hypothesis tool use arises due to genetic changes that cause an increase in the capacity for problem-solving [9,12]. Selection for these cognitive capacities is then reinforced through the construction of tools that mitigate environmental constraints and increase fitness [10,13]. Others have argued that the selection of specific cognitive traits is not necessary as social learning can facilitate the accumulation of information across generations [11,14]. While this process is particularly potent in humans [14], population-scale cultural processes driven by social learning have also been argued to produce diverse behavioural patterns in primate populations [15–19].

As a result, there has been an increasing focus on identifying the mechanisms that non-human primates use to transfer skills between individuals to determine if some share evolutionary roots with those employed by humans [12,18,20–23]. Extensive work on various primate species, including chimpanzees (*Pan troglodytes*), macaques (*macaca fascicularis*) and capuchins (*Sapajus libidinosus*), indicate that the acquisition and transmission of tool-using behaviours are socially mediated [5,24–30]. Naive individuals often generate observational opportunities for themselves by maintaining proximity to tool users or showing interest in the residual materials associated with behaviour [24,25,31,32]. For example, mothers often provide their offspring with observational learning opportunities critical for the acquisition of skills [31,33–35]. On the other hand, certain genetic predispositions are argued to provide the biological basis necessary for tool use [12,13]. For example, genetic evidence of chimpanzees has shown that general factors that influence the capacity to use tools such as problem-solving, motor skills and social aptitude are heritable [36,37]. However, whether the presence of a behavioural trait within a population is due to social or genetic factors is often obvious at the species level it is more difficult to discern within an individual species. Within a single primate species, the studies of transmission mechanisms often rely on between-group comparisons where researchers determine the influence of a transmission mechanism by attempting to rule out all other factors. However, between-group comparisons of various primate species are often confounded by the influence of ecology on observed patterns [22,38,39]. Moreover, group comparisons investigating whether behaviour variation is due to genes or culture have produced contradictory results even when the same datasets are used [13,20,22]. For example, two studies that examined behavioural variation within *Pan* to determine that between-group behavioural variation was the result of cultural processes while the other suggested that these differences were due to genetics [20,22].

Hybrid populations provide a rare opportunity to investigate the social and/or genetic drivers of behavioural variation in a single ecological setting [40–42]. The long-tailed macaques of Koram Island, Thailand are a cross between, the tool-using Burmese (*Macaca fascicularis aurea*) and non-tool-using common (*Macaca fascicularis fascicularis*) sub-species [13]. The Burmese sub-species habitually use stone tools to forage for marine resources, including crabs, snails and oysters [43–45]. By contrast, individuals from the common sub-species use tools in neither wild nor captive settings despite prolonged exposure to resources [13,46]. While the Koram population also uses tools to forage for a similar array of marine resources, observations suggest that variation in whether individuals use tools may be linked to differences in genetic contributions from each sub-species [13]. Previous research revealed that only about half of the Koram population were tool users; a substantially lower proportion of tool users than what is observed in a pure Burmese population [13]. A comparison of tool use frequency and phenotype found that individuals who displayed more Burmese-like features (hybrid-like) were significantly more likely to be tool users than those who displayed common-like phenotypes (see [13] for how the phenotype is determined). This work has led to the suggestion that traits conducive to acquiring tool use might be genetically inherited [13].

Observational studies have also provided evidence for socially mediated learning of tool skills [32,34]. Tool use emerges in the Koram individuals after a 3-year associative process [32]. A few months after birth, individuals will begin engaging with tool materials [32]. Over the following 2 years, initial object manipulation gives way to combining objects associated with tool use and eventually using them in percussive actions. Individuals continue to improve in skill and efficiency as they increase in age [32]. Analysis of grooming patterns suggests younger individuals will preferentially associate with older, more skilled tool users throughout this developmental period, further suggesting that social learning may play a role in the acquisition of tool use [34]. These macaques therefore provide the rare opportunity to study the social and inherited determinants of tool use within a single wild population of primates.

Social network analysis (SNA) allows us to characterize and quantify the associative relationships between individuals necessary for understanding information transmission patterns [47–49]. Such studies have successfully combined data regarding associative relationships with the appearance of behaviours in individuals to infer the information transmission mechanism influencing a population [28,29,50–52] For example, network-based diffusion analyses combine social network analysis with data regarding the time and order of when specific individuals first exhibit a behaviour within a population [51,52]. Despite the utility of SNA, the application of such analyses to wild primate populations requires multi-generational data that can take years if not decades to amass [53]. In many cases, observational data of primate behaviour comprise only one or two generations of associations between individuals and thus lack the longitudinal depth required for network-based diffusion analysis. Therefore, methods that allow researchers to investigate information transmission processes in short-term datasets are needed. In order to develop such methods, however, it is critical to understand how various information transmission mechanisms influence the associations between knowledgeable and naïve individuals within a social network,

Agent-based modelling (ABM) provides an investigative framework through which various processes can be simulated and their influence on social network patterns can be explored. The generative nature of ABM allows us to translate the dynamics associated with various mechanisms of information transfer into datasets that can be analysed in the same way that real-world data are investigated. Therefore, the outcomes of the ABM can be used to develop tangible expectations for how various mechanisms of information transfer can be detected in real-world datasets. While these expectations do not allow us to make explicit inferences, they do provide an informed and investigative framework for discussing the potential roles of various information transmission processes in a living population.

Here, we investigate how inheritance and social transmission processes potentially influence the associations between tool users and non-tool users within a group of long-tailed hybrid macaques on Koram Island. We develop an ABM to explore how genetic inheritance, social transmission and asocial transmission processes would structure the associations between tool users and non-tool users within a social network. The social learning conditions of the ABM assume that naive individuals can only acquire tool use when they are in proximity to a tool user. Whereas, under inheritance conditions, tool use could only be gained via transmission from parents to offspring during reproduction. Under asocial conditions tool use is acquired independently. The associations between tool users and non-tool users elucidated by the models provide useful expectations for how various information transmission mechanisms impose patterning on a social network. We then discuss the associations between tool users and non-tool users observed in the hybrid macaque group on Koram Island in light of the outcomes of ABM. The results of this work, thus, provide a context by which to investigate the underlying mechanisms involved in the acquisition of tool use.

## 2. Study population

**Figure 1.**
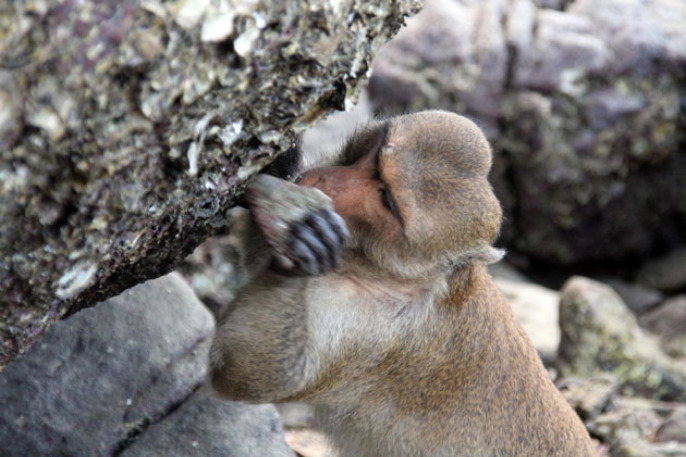
Male macaque using a stone hammer to forage on rock oysters (Photo credit: Amanda Tan).

Koram Island is situated about 1 km off the shore of the Gulf of Thailand within Khao Sam Roi Yot National Park, Thailand. The island possesses about 3.5 km of coastline consisting of rocky shores with small sandy beaches where the macaques engage in tool-assisted foraging of marine invertebrates at low tide (figure 1). Over 227 days between 17 October 2013 and 2 December 2014, focal data were collected on 69 individuals from a single group. Focal follow data were collected during a daily 4 h window during low tide when marine invertebrates are accessible for foraging. Individuals were continuously sampled in random order at 5 min intervals culminating in a total 499 of hours of observation and 5990 focal scans. Author AT generated lists of individuals in randomized order and focal samples of individuals were conducted according to these lists. All data were recorded by narrating observations into a voice recorder and then transcribing into excel. During each focal, AT recorded data on tool use behaviour including when individuals picked up and dropped tools, the starts, and ends of tool use bouts, the number of strikes and hammering pattern applied, the type of food processed, and whether the bout was successful. AT also recorded social data including when the focal individual entered and exited a 1 m radius of other individuals and the identities of these individuals, as well as the starts and ends of grooming bouts and identities of grooming partners. These data enable us to determine tool user status for all mature individuals (*n* = 42) and to construct social networks.

In 2015, AT collected photographic data of the 42 individuals to assess their facial pelage patterns and assign them to a phenotypic category—common-like or hybrid-like [13,43]. Facial pelage has been shown to be an accurate proxy of phenotype as studies across Southeast Asia indicate that features of facial pelage associated with the two sub-species are distinct [54]. Hybrid individuals are recognized by the presence of varying combinations of common and Burmese features [55]. The hybrid phenotype is more similar to the tool-using Burmese long-tailed pattern than the common phenotype implying a greater contribution of Burmese ancestry (See Gumert *et al*. [13], for a detailed description). To determine the phenotypic category, AT collected photos of the front sides of each individual's face, and an independent rater classified them while blind to the individual's tool use ability.

## 3. Analytical methods

### (a) Characterization of associations during foraging

To investigate the role of various transmission mechanisms on tool use acquisition, associative relationships between tool users and non-tool users during foraging bouts were described using social network analysis [47]. Thus, a directed social network was constructed and quantified based on 1 m proximity associations of Koram individuals during foraging using the igraph package (v. 1.3.4) in R (v. 4.1.2), [56,57]. Connections between individuals were weighted by the number of times they were observed within 1 m of each other. A directed social network was chosen because each association is comprised of an individual engaging in foraging activity and an individual within a meter of the active individual. Therefore, the assumed transfer of information is from the active individual to the individual within 1 m.

The position of each individual within the network was characterized by calculating each individual's eigenvector centrality (EV-centrality) score [49]. EV-centrality is a commonly used measure to identify individuals who are most connected within a social group [28,48,58]. This measure has been proven to be important regarding social learning as individuals at the centre of primate social networks are more knowledgeable and more likely to learn novel behaviours [28,58]. EV-centrality is calculated as the sum of centralities the of an individual's neighbours [49]. Individuals at the centre of a given network have more connections with other individuals and thus have higher EV-centrality values. In addition, since social learning requires repeat encounters with tool users, each individual was also characterized in terms of their connection strength to tool users (strength). Connection strength characterizes how strong an individual's connection is to another individual [59]. Connection strength to tool users was calculated by summing the weight of each connection that an individual has with the tool-using individuals.

#### (i) Generating expectations for information transmission mechanisms

To place the Koram network analysis within a broader interpretive framework, we developed three ABMs to generate expectations for how social, inheritance or asocial learning mechanisms produce measurable patterns within a social network. The ABMs were developed in Python 3.9 using the ABM python library MESA [60,61]. The full description of the ABM is provided as electronic supplementary material, and the code is actively maintained on the author's GitHub page (see https://github.com/reevesj191/Macaque_Tool_Transmission) [62].

### (b) General overview of the agent-based model environment

While the information transmission mechanisms vary between models, the general conditions of each model are the same. Each model consists of 100 agents that move through a 20 × 20 grid cell space, in a random fashion. When the model is instantiated 100 agents are randomly distributed onto the grid space. To match the measured attributes, present within the Koram dataset, agents possess the attributes tool user status, phenotype and age. Tool user status corresponds to whether the agent possesses the ability to use tools. Of the initial 100 agents, 99 are classified as non-tool users. Phenotype is randomly assigned as either ‘hybrid-like’ or ‘common-like’ to reflect the phenotypic categories within the Koram population. Age is initially assigned by randomly drawing a number between 0 and 100. Age does not equate to years but rather the number of time steps an individual has existed in the grid space. We assign a random number to the age of the initial agents in the simulation to prevent mass die-off events during the simulation. The single remaining agent is given tool user status, the hybrid-like phenotype and an age of 25 time steps. To consider that, within the Koram population, tool use is acquired as a part of a multi-year developmental process agents with an age of 25 or less cannot acquire the tool use trait.

During each time step, each agent moves a unit of 1 time step by randomly choosing one of its neighbouring grid cells. Agents then have the opportunity to acquire tool use according to the conditions of one of three modelled transmission modes (See below). Each simulation runs until 50% of the population gains the tool use skill, the proportion of tool users present within the Koram macaques [13].

### (c) Models transmission mechanisms

#### (i) Social learning model

Under conditions of the social learning model, the tool use trait is only acquired through interactions with agents who already possess the tool user trait. During each time step agents have a chance to acquire tool user status if they move into a grid cell occupied by a tool-using agent. The likelihood that an agent becomes a tool user is determined based on the number of previous encounters with tool users. Each time a naïve agent encounters a tool user, the likelihood that they acquire the trait increases by 1%. While 1% appears to be a small increase, this value was chosen given that the Koram macaques acquire tool use over a 3-year associative process. Therefore, a 1% increase is appropriate given the rate at which the Koram macaques learn tool use in the wild. Thus, as an agent accrues proximity interactions with tool users, the likelihood of skill acquisition increases.

#### (ii) Genetic inheritance model

Under conditions of genetic inheritance, the tool use trait is passed from parent to offspring via reproduction. Therefore, additional parameters governing reproduction, ageing, death and population size are defined. In addition to the general parameters outlined above, the age of agents increases by a unit of 1 during each time step. The age of the agent corresponds to the number of time steps the agent has existed in the grid space. Then, the agent will determine if it lives or dies. Whether the agent dies is determined by drawing from a binomial distribution with a baseline death probability of 0.0001. This baseline is further weighted by the age of the agent by adding the age of the agent divided by 0.00001. Therefore, the likelihood of death is kept at any given time step is low. The death probability is kept intentionally low to ensure that agents remain alive long enough to have a chance to reproduce with another agent.

Agents can also reproduce when they share a grid cell with another agent, creating a new agent with an age of 0. Reproductive events occur when two agents share the same grid cell. During this, a new agent (the ‘offspring’) is created at the same location as the parent agents. The offspring's phenotype is inherited from the parents using the following system: if both parents possess the same phenotype (i.e. ‘hybrid-like’ or ‘common-like’), then the offspring agent will be assigned this phenotype. If the parents' phenotypes differ from each other, then the offspring's phenotype is assigned based on a random choice. To explicitly model the notion that phenotype predicts tool use—as is suggested by Gumert et al. [13]—the tool use trait is only inherited when the ‘hybrid-like’ phenotype is also inherited.

Finally, to consider the influence between mothers, their offspring and the acquisition of tool use, newly born agents move depending on their mother's location. As offspring increase in age, the influence of the mother's location on their movement lessens and their movement eventually becomes random (as defined above). Within the simulation, agents preferentially pick the neighbouring grid cell that minimizes the distance from their mother. The probability that agents chose this grid cell is equal to two times and age of the agent divided by 100. This ensures agents will almost always follow their mother agents when they are very young but less so when they are older.

#### (iii) Asocial model

Given that the Koram data are comprised of proximity associations during foraging, it is important to rule out the influence of ecological processes on the structure of the social network. For example, locations of tool-required resources could cause tool users to occupy specific locations within the wider foraging landscape. Such a phenomenon could cause non-random associations between tool users and non-tool users simply due to a preference for specific locations as opposed to information transmission. Thus, within the asocial model agents are given a five per cent chance to independently acquire the tool use trait after every time step. Moreover, to examine the effect of spatial preference, agents with the tool use trait will preferentially, according to a user-defined probability, move toward the location of ‘attractors’ that are randomly distributed within the grid space. The chance that a tool-using agent will move towards an attractor was set to 25%. Furthermore, the influence of resource attraction on the preferential association of tool users is likely dependent on the number of resources that require tool use to gain access. If the foraging landscape possesses a single location where tool use is required, then we expect a strong association between tool users because they will all gravitate to a single location. However, if resources are abundant and widespread, the effect of resource attraction on the number of associations between tool users might be weak. To examine this relationship, we varied the number of ‘attractors’ present also varied between 1, 10 and 200.

#### (iv) Generative output

At the end of each simulation, a record of proximity associations between agents is outputted. An association is defined as any time two agents share the same grid cell. In addition, agent-specific information including, age, phenotype and tool user status is also exported. These data are then analysed in the same fashion as the observed data, allowing the ABM data to be investigated in the same manner as the data collected from Koram.

### (d) Statistical analysis

To investigate the outcomes of the data generated by the ABM with the observed data from Koram Island, we developed a binomial linear model with a logit function to test the effects of phenotype, age and foraging associations on tool user status. In addition to the data from Koram Island, this statistical model is also applied to data generated from 30 iterations of each condition of the ABM. By applying the same statistical model to both datasets, we gain insight into how the variables age, phenotype, EV-centrality and strength influence the tool use status under various conditions of information transfer. In doing so, it becomes possible to assess the roles of social learning, inheritance and asocial processes in the acquisition of tool use within the Koram macaques. Code, equations and measures of performance associated with this model are provided as electronic supplementary material and available on the author's GitHub page (see https://github.com/reevesj191/Macaque_Tool_Transmission).

The response variable is defined as tool user status. To examine the effect of phenotype on tool user status, phenotype was included as a categorical variable. Moreover, to assess the effect of social network position on tool user status, EV-centrality and strength were included as continuous variables. Age was also included in the model as a control variable given that tool use is only observed in individuals after 2.5–3.5 years. To do so, age was broken down into the categories of developmental age and tool user age, and included as interaction with phenotype, EV-centrality and strength. We used uninformed regularizing priors to ensure the model did not produce unrealistic outcomes. Priors for age and phenotype were represented by a normal distribution with a mean of 0 and a s.d. of 1. The prior for centrality is modelled as lognormal distribution as previous studies show that increasing centrality increases the likelihood of receiving new information [28]. Similarly, the prior for strength was also included as a Lognormal distribution as individuals that are more strongly connected to tool users will be more likely to acquire tool use as a skill. The model was fitted to each of the simulated conditions of asocial learning, social learning and inheritance as well as the data from Koram Island using the Hamiltonian Monte Carlo engine in Stan v. 2.28.1 [63] using the rethinking package in R v. 4.1.2 [64].

## 4. Results

### (a) Simulations

**Figure 2.**
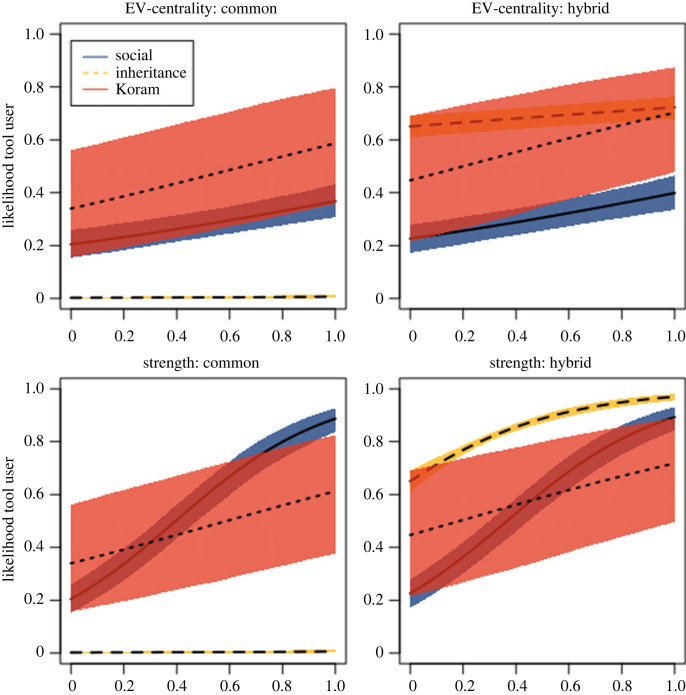
Posterior predictive plots illustrating the effect of social network position (EV-centrality and strength on the *x*-axis) on tool user status according to phenotype (in individuals old enough to be able to use tools) under simulated conditions of social learning and inheritance, alongside the Koram Island macaques. Note that there is a wide range of uncertainty associated with the data from Koram Island. Nevertheless, the social learning simulation falls within the 89% compatibility interval surrounding the observed data from Koram Island. Whereas the inheritance simulation generally falls outside of this compatibility interval.

**Figure 3.**
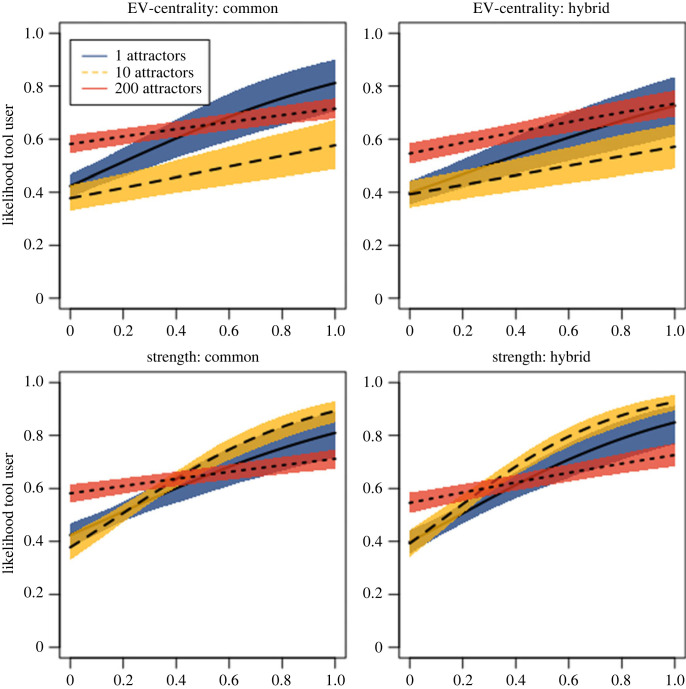
Posterior predictive plots illustrating the effect of social network position (EV-centrality and strength on the *x*-axis) on tool user status according to phenotype (in individuals old enough to be able to use tools) under model asocial conditions. Note that the pattern associated with the asocial model is heavily influenced by the number of attractors included in the model.

Summaries of the marginal distributions of each parameter under each simulated condition are presented in tables 1 and 2. However, each parameter cannot be directly interpreted as to its importance in determining the likelihood that an individual is a tool used due to the interactions between the variables. Instead, these values should be viewed in combination with the posterior predictions visualized in figures 1–3. A full summary of the diagnostics and visualizations can be found on the author's GitHub page (see https://github.com/reevesj191/Macaque_Tool_Transmission).

**Table 1.** Marginal distributions for each Bayesian model associated with the social learning and genetic inheritance ABM conditions and the Koram data.

	mean	s.d.	5.50%	94.50%	*N* effective samples	\hat{\bi R}
**ABM social learning**						
intercept (DA, common like)	0.002	0.645	0.001	0.004	3078	1
intercept (DA, hybrid)	0.003	0.656	0.001	0.007	3448	1
intercept (adult, common)	0.216	0.547	0.169	0.271	2768	1
intercept (adult, hybrid)	0.251	0.552	0.193	0.314	3007	1
centrality (DA, common)	0.708	0.561	0.633	0.792	4253	1
centrality (DA, hybrid)	0.708	0.563	0.631	0.790	4115	1
centrality (adult, common)	0.668	0.542	0.612	0.729	3598	1
centrality (adult, hybrid)	0.689	0.549	0.628	0.756	3470	1
strength (DA, common)	0.717	0.566	0.637	0.798	3840	1
strength (DA, hybrid)	0.717	0.564	0.640	0.800	4170	1
strength (adult, common)	0.972	0.586	0.953	0.984	2886	1
strength (adult, hybrid)	0.958	0.592	0.927	0.977	3914	1
**ABM genetic inheritance**						
intercept (DA, common like)	0.007	0.659	0.002	0.019	4240	1
intercept (DA, hybrid)	0.006	0.654	0.002	0.015	4571	1
intercept (adult, common)	0.002	0.646	0.001	0.006	4246	1
intercept (adult, hybrid)	0.679	0.533	0.630	0.723	2801	1
centrality (DA, common)	0.738	0.573	0.653	0.824	4892	1
centrality (DA, hybrid)	0.739	0.581	0.649	0.837	4292	1
centrality (adult, common)	0.733	0.575	0.647	0.827	3582	1
centrality (adult, hybrid)	0.599	0.521	0.568	0.634	5042	1
strength (DA, common)	0.736	0.578	0.648	0.828	4872	1
strength (DA, hybrid)	0.736	0.576	0.651	0.826	4648	1
strength (adult, common)	0.725	0.569	0.643	0.813	4739	1
strength (adult, hybrid)	0.928	0.597	0.874	0.961	2723	1
**Koram Island**						
intercept (DA, common like)	0.199	0.751	0.040	0.591	3902	1
intercept (DA, hybrid)	0.082	0.716	0.019	0.260	2966	1
intercept (adult, common)	0.328	0.647	0.158	0.562	3334	1
intercept (adult, hybrid)	0.442	0.657	0.215	0.691	2534	1
centrality (DA, common)	0.733	0.576	0.647	0.825	3697	1
centrality (DA, hybrid)	0.731	0.574	0.647	0.823	3721	1
centrality (adult, common)	0.750	0.581	0.658	0.844	3057	1
centrality (adult, hybrid)	0.763	0.594	0.662	0.862	2871	1
strength (DA, common)	0.740	0.583	0.647	0.837	3277	1
strength (DA, hybrid)	0.741	0.580	0.651	0.836	3747	1
strength (adult, common)	0.772	0.597	0.669	0.872	3404	1
strength (adult, hybrid)	0.778	0.600	0.672	0.880	2525	1

**Table 2.** Marginal distributions for each Bayesian model associated with the asocial learning ABM conditions.

	mean	s.d.	5.50%	94.50%	*N* effective samples	\hat{\bi R}
**ABM asocial: number of attractors 1, attractor strength 25**						
intercept (DA, common like)	0.003	0.649	0.001	0.008	3457	1
intercept (DA, hybrid)	0.004	0.651	0.001	0.010	3469	1
intercept (adult, common)	0.422	0.527	0.379	0.463	2508	1
intercept (adult, hybrid	0.399	0.528	0.357	0.442	2552	1
centrality (DA, common)	0.729	0.571	0.646	0.816	3230	1
centrality (DA, hybrid)	0.729	0.573	0.643	0.822	3580	1
centrality (adult, common)	0.861	0.596	0.769	0.920	2844	1
centrality (adult, hybrid)	0.806	0.584	0.713	0.880	2392	1
strength (DA, common)	0.727	0.570	0.646	0.814	3296	1
strength (DA, hybrid)	0.726	0.570	0.644	0.811	3340	1
strength (adult, common)	0.858	0.600	0.764	0.922	1879	1
strength (adult, hybrid)	0.898	0.602	0.821	0.944	1998	1
**ABM asocial: number of attractors 10, attractor strength 25**						
intercept (DA, common like)	0.004	0.649	0.001	0.009	4363	1
intercept (DA, hybrid)	0.003	0.654	0.001	0.008	3128	1
intercept (adult, common)	0.386	0.533	0.338	0.437	2258	1
intercept (adult, hybrid)	0.408	0.533	0.359	0.460	2528	1
centrality (DA, common)	0.728	0.572	0.644	0.817	4356	1
centrality (DA, hybrid)	0.724	0.571	0.640	0.813	4774	1
centrality (adult, common)	0.689	0.542	0.633	0.747	3208	1
centrality (adult, hybrid)	0.693	0.546	0.630	0.755	3483	1
strength (DA, common)	0.724	0.572	0.639	0.816	4054	1
strength (DA, hybrid)	0.724	0.569	0.643	0.811	4343	1
strength (adult, common)	0.933	0.584	0.890	0.960	2085	1
strength (adult, hybrid)	0.929	0.589	0.879	0.959	2189	1
**ABM asocial: number of attractors 200, attractor strength 25**						
intercept (DA, common like)	0.003	0.648	0.001	0.007	3578	1
intercept (DA, hybrid)	0.003	0.650	0.001	0.008	2827	1
intercept (adult, common)	0.587	0.521	0.553	0.619	3312	1
intercept (adult, hybrid)	0.579	0.522	0.544	0.611	3348	1
centrality (DA, common)	0.721	0.570	0.639	0.808	3408	1
centrality (DA, hybrid)	0.723	0.569	0.640	0.811	3854	1
centrality (adult, common)	0.646	0.531	0.603	0.691	3515	1
centrality (adult, hybrid)	0.647	0.532	0.603	0.695	3955	1
strength (DA, common)	0.720	0.569	0.637	0.808	3810	1
strength (DA, hybrid)	0.722	0.569	0.639	0.809	3386	1
strength (adult, common)	0.644	0.532	0.600	0.693	3578	1
strength (adult, hybrid)	0.650	0.534	0.602	0.701	3155	1

As expected, the effect of age is consistent across all information transmission conditions. Recall that the ABM explicitly prevents the agent under the age of 25 from acquiring the tool use trait. Therefore, the likelihood that an individual is under the minimum age necessary to acquire tool use is zero. For individuals who are of age to learn to use tools, the variables that influence the likelihood of tool user status are dependent on the mode of information transmission. Under conditions of social learning, both strength and centrality are important factors in determining tool user status. The average tool user possessed greater strength values than non-tool users in 97% of the model iterations. In terms of EV-centrality, the average tool users possessed greater centrality values than non-tool users in 77% of the iterations. Strength has the strongest positive effect on the likelihood that an agent is a tool user (figure 2). Individuals that establish stronger connections with tool users are more likely to become tool users than those who do not. Moreover, EV-centrality also has a positive effect on the likelihood that an individual is a tool user. Individuals that hold central positions are those that have established connections with many other individuals and are therefore more likely to have encountered tool users. However, the effect of EV-centrality is substantially milder when compared to strength. On the other hand, Phenotype has no influence on tool user status under social learning conditions. This is also to be expected, given that agents do not bias in who they affiliate with according to the phenotype variable. There is little difference in the likelihood that an individual is a tool user when the phenotype is considered (figure 2).

In contrast with the social learning condition, phenotype has the greatest effect on tool user status under conditions of inheritance. Agents with a ‘hybrid-like’ phenotype have a 60% chance of being a tool user, whereas those with a ‘common-like’ phenotype have zero chance. Again, this is to be expected as tool use can only be inherited from individuals with a ‘hybrid-like’ phenotype. In addition, there is very little difference between the centrality scores of tool users and non-tool users with the average difference between tool users and non-tool users being 0.01. As a result, centrality has a marginal effect on tool user status under conditions of genetic inheritance (figure 2). This is because pure inheritance decouples the acquisition of the tool use skill from social encounters with other tool users. Although it is less prominent than what is observed in the social learning model, strength still has a positive effect on tool user status. This effect is driven by the spatial associations between new offspring agents and tool-using mothers. Given that offspring attempt to minimize the distance from their mothers, offspring that inherit tool user status will preferentially associate with tool users, thus increasing their connection strength with tool users.

The results of the asocial model indicate that a mere attraction to specific locations can have a strong effect on the relationship between tool users and non-tool users within a social network (table 2). When there is a single attractor the asocial model both measures of strength and EV-centrality have a strong positive effect on the likelihood of tool user status (figure 3). This is due to the fact that the attraction to a single or few places in the landscape causes tool users to aggregate at the same locations and are, thus, more likely to associate with each other than non-tool users. However, as the number of preferred locations increases, this effect diminishes as the increasing number of attractors ensures that tool users do not spend all their time in a single part of the grid space. The positive effect of strength and EV-centrality on tool user status is reduced to a marginal influence as the proportion of attractors reaches 50%.

#### (i) Observational data of the Koram macaques

When the observed data from Koram are considered, age has a similar effect on the likelihood that an individual is a tool user when compared with all of the model results. Individuals younger than the age at which tool use typically emerges (infants and juveniles) are unlikely to be tool users regardless of their social network position or phenotype (table 1). This is to be expected given the multi-year process over which tool use emerges [49]. However, the observed data reveal a negative effect on the likelihood that an individual becomes a tool user that is not as severe as the effects reported in the simulated data (figure 2). This is likely because there is a time window in which tool use emerges within the Koram individuals as opposed to the set age at which tool use appears. Therefore, within the Koram data, a few individuals may acquire tool use at an earlier age.

**Figure 4.**
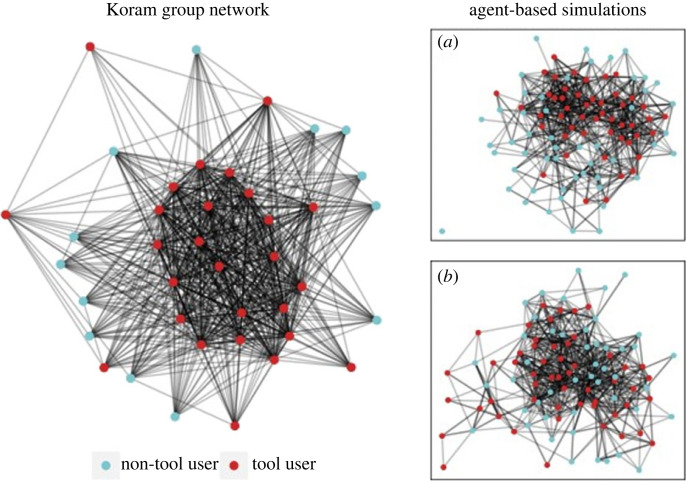
The Koram social network compared to examples of social networks generated by the simulated conditions of social learning and inheritance. The Koram social network is based on 1 m proximity data during foraging. That tool users (displayed in red) hold more central positions than non-tool users within the Koram network. The insets (*a*,*b*) show examples of simulated social networks under different conditions of information transfer. The layout of the social networks is force-directed, nodes that are closer to each other share more connections with each other than those that are farther away. (*a*) Social learning condition: an example of a simulation where tool use is transmitted through social learning. Note that tool users are more centrally clustered than non-tool users. (*b*) Genetic inheritance condition: an example of a simulation where tool use is transmitted through inheritance. Note that there is less of a structured relationship between tool users and non-tool users.

Among individuals of tool-using age, centrality and strength, both have a positive effect on the likelihood that an individual is a tool user (figures 2 and 4). Individuals who hold more central locations and are more strongly connected with other tool users within the social network are, thus, more likely to be tool users than those on the periphery (figure 4). Phenotype also influences tool user status among the Koram macaques. Individuals with a hybrid-like phenotype are 11% more likely to become tool users than those possessing a common-like phenotype. However, this is particularly the case when individuals hold more central positions in the social network. The increased positive effect of possessing a hybrid-like phenotype on tool use falls within the 89% compatibility interval associated with the common-like phenotype.

## 5. Discussion

Understanding the pathways through which primates acquire tool use is an important facet of cultural evolution research. Yet, inferring the mechanisms by which wild primates acquire skills remains difficult. The integration of agent-based simulation with a Bayesian linear model provides expectations for how social learning, asocial and genetic inheritance processes can be distinguished using social network data. Under conditions of social learning individuals that hold more central positions within the network and have stronger connections to other tool users are more likely to be tool users themselves. Under conditions of pure genetic inheritance, neither centrality nor strength will have a positive influence on an individual's status as a tool user. If phenotype and the genetic inheritance of tool use are linked, as they are in the ABM, it should be expected that individuals carrying the ‘hybrid-like’ phenotype will have a positive effect on tool user status. Our results also show how food resource distribution can produce preferential associations between tool users. Nevertheless, when food resources are abundant, connection strength to tool users and EV-centrality have little effect on the likelihood that an individual becomes a tool user. These results demonstrate that the statistical model we applied is effective in detecting differences in datasets generated by the three model conditions.

When the social network patterning in the Koram population is compared to the ABM results, a number of consistencies and inconsistencies are observed. These similarities and differences provide an interpretative context through which the influence of information transmission processes on skill acquisition, within a living population, can be discussed. When the Koram macaques are considered, the positive effect of EV-centrality and strength on tool user status is similar to what is predicted by the social learning model. This finding is also consistent with observations of the associative learning process through which tool use emerges within the group [32]. In comparison with the social learning model, however, the observed effect of strength on tool user status is less strong, whereas the effect of EV-centrality is stronger. The positive effects of EV-centrality and strength on tool user status are also consistent with the asocial model, particularly when the number of fixed resources is few. At face value, the similarities with the asocial learning model may suggest that the pattern observed within the Koram population could simply reflect a preference for tool users to occupy specific places during foraging. However, when the abundance resources on of Koram Island is considered, it is clear that this is not the case. The shores on which the Koram macaques forage are rich in marine resources implying that there is an abundance of locations where tools can be used. Moreover, while oysters are sessile upon a fixed substrate (e.g. rocks and boulders), they are distributed continuously along the rocky shore. In addition, many other marine resources (snails and crabs) that the macaques forage on will change location over time. Thus, it is unlikely that the distribution of resources requiring tool use would cause tool users to preferentially associate with one another simply due to the distribution of resources. Therefore, we argue that the positive effects of EV-centrality and Strength are most likely related to social learning processes.

Research by Gumert *et al*. [13] shows that 76% of hybrid-like phenotypes are tool users whereas 42% of common-like phenotypes are tool users. This may suggest that some prerequisite components needed for tool use are possibly inherited. Our results maintain some level of uncertainty surrounding the effect of the hybrid-like phenotype on tool user status (figure 2). Nevertheless, there is reason to suggest that this effect is biologically meaningful. Previous research on Burmese long-tails (*Macaca fascicularis aurea*) shows that almost 90% of individuals within a single population are tool users [13]. These observations show that the higher proportion of tool users observed in the hybrid-like individuals is more consistent with the proportion of tool users observed in Burmese populations. Within the broader context of primate behaviour, social learning is understood to play a major role in tool use acquisition [65]. Usually, all members of tool-using primate groups are tool users [15]. Thus, if social learning were entirely responsible for the prevalence of tool use on Koram Island, we would expect there to be a greater proportion of tool users within the population. Instead, only half of the population exhibits the behaviour, the majority of those expressing the hybrid phenotype. Inheritance therefore might play a role in providing the necessary conditions required for tool use. Our work suggests that the actual acquisition of the skill, however, is facilitated through mechanisms of social learning but is not sufficient to explain tool use in the Koram macaques on its own.

Additional support for this notion could be found by examining mother–offspring relationships. Mothers are an important source of observational opportunities for younger individuals. Therefore, under conditions of social learning, the tool use preferences of offspring should reflect those of their mother. On Koram island, however, where mother–offspring relationships are known for individuals old enough to use tools, there are at least two instances where the tool user status of the mother is not the same in the offspring. In one instance, a tool-using mother raised an individual that did not become a tool user. In another, it was the other way around. Though these data are limited, they suggest that offspring may not always mirror the behaviours of their mother.

It is most likely that social and inheritance factors both contribute to the acquisition of tool use within this population. It has already been shown that certain motor control and acquisition of socially learned behaviours in primates are highly heritable [36,37,66]. While all behaviours involving tool use are likely mediated by a combination of inherited and social factors [67,68], the hybrid long-tailed macaques of Koram may be a unique case where inheritance still dictates whether an individual can acquire tool use as a skill within one population. While the simulations presented here allow us to generate expectations under conditions of inheritance, the genetic processes that govern inheritance are likely far more complex in the real world. Further genetic studies therefore can help investigate potential predispositions regarding tool use. This information would help to further entangle requirements that are necessary for the acquisition of tool use, contributing to ongoing discussions regarding the evolution of tool use in human and non-human primates [2,9–11,14,46,69]

Within the context of broader evolutionary theory, hypotheses favouring the selection of cognitive traits that facilitate tool use are often pitted against those that favour cultural explanations as competing hypotheses [9,10,70]. The results of our work suggest that both inheritance and social processes play an active role in the prevalence of tool use within long-tailed macaques. This further suggests that social processes and predispositions need not be mutually exclusive. Thus, these hybrid macaques may be a living example of how predispositions mitigate the acquisition of a socially learned behaviour. Such learned behaviours in return may result in the selection of cognitive traits or developmental biases that further promote the prevalence of tool use within a population. The combination of applying ABM and social network analysis to technological primates provides a novel opportunity to investigate the role of these underlying processes on tool use prevalence.

## Ethics

Research in the Kingdom of Thailand and Khao Sam Roi Yot National Park was approved by the National Research Council of Thailand (NRCT), and the Department of National Parks, Wildlife, and Plant Conservation. Research methods were approved by the Nanyang Technological University (NTU), Institutional Animal Care and Use Committee (ARF SBS/NIE-A 0210 AZ).

## Data Availability

Data are available on Dryad [71] and on the author's GitHub page at https://github.com/reevesj191/Macaque_Tool_Transmission. Additional information is provided in the electronic supplementary material [72].

## References

[1] Hill K, Barton M, Magdalena Hurtado A. 2009 The emergence of human uniqueness: characters underlying behavioral modernity. Evol. Anthropol. **18**, 187-200. (10.1002/evan.20224)

[2] Whiten A, Caldwell CA, Mesoudi A. 2016 Cultural diffusion in humans and other animals. Curr. Opin. Psychol. **8**, 15-21. (10.1016/j.copsyc.2015.09.002) 29506791

[3] Lotem A, Halpern JY, Edelman S, Kolodny O. 2017 The evolution of cognitive mechanisms in response to cultural innovations. Proc. Natl Acad. Sci. USA **114**, 7915-7922. (10.1073/pnas.1620742114) 28739938 PMC5544270

[4] Laland KN, O'Brien MJ. 2011 Cultural niche construction: an introduction. Biol. Theory **6**, 191-202. (10.1007/s13752-012-0026-6)

[5] Bandini E, Tennie C. 2020 Exploring the role of individual learning in animal tool-use. PeerJ **8**, e9877. (10.7717/peerj.9877) 33033659 PMC7521350

[6] Caldwell CA, Millen AE. 2009 Social learning mechanisms and cumulative cultural evolution: is imitation necessary? Psychol. Sci. **20**, 1478-1483. (10.1111/j.1467-9280.2009.02469.x) 19891752

[7] Whiten A, Horner V, Marshall-Pescini S. 2003 Cultural panthropology. Evol. Anthropol. **12**, 92-105. (10.1002/evan.10107)

[8] Luncz LV, Wittig RM, Boesch C. 2015 Primate archaeology reveals cultural transmission in wild chimpanzees (*Pan troglodytes verus*). Phil. Trans. R Soc. Lond. B **370**, 20140348. (10.1098/rstb.2014.0348) 26483527 PMC4614712

[9] Pinker S. 2010 The cognitive niche: coevolution of intelligence, sociality, and language. Proc. Natl Acad. Sci. USA **107**, 8993-8999. (10.1073/pnas.0914630107) 20445094 PMC3024014

[10] Morgan TJH. 2016 Testing the cognitive and cultural niche theories of human evolution. Curr. Anthropol. **57**, 370-377. (10.1086/686531)

[11] Boyd R, Richardson PJ. 2005 Not by genes alone: how culture transformed human evolution. Chicago, IL: University of Chicago Press.

[12] Call J. 2013 Three ingredients for becoming. In Tool use in animals: cognition and ecology (eds CM Sanz, J Call, C Boesch), pp. 3-20. Cambridge, UK: Cambridge University Press.

[13] Gumert MD, Tan AWY, Luncz LV, Chua CT, Kulik L, Switzer AD, Haslam M, Iriki A, Malaivijitnond S. 2019 Prevalence of tool behaviour is associated with pelage phenotype in intraspecific hybrid long-tailed macaques (*Macaca fascicularis aurea*×*M. f. fascicularis*). Behaviour **156**, 1083-1125. (10.1163/1568539X-00003557)

[14] Henrich J. 2017 The secret of our success. Princeton, NJ: Princeton University Press.

[15] Whiten A, Goodall J, McGrew WC, Nishida T, Reynolds V, Sugiyama Y, Tutin CEG, Wrangham RW, Boesch C. 1999 Cultures in chimpanzees. Nature **399**, 682-685. (10.1038/21415) 10385119

[16] van Schaik CP. 2003 Orangutan cultures and the evolution of material culture. Science **299**, 102-105. (10.1126/science.1078004) 12511649

[17] Ottoni EB, Izar P. 2008 Capuchin monkey tool use: overview and implications. Evol. Anthropol. **17**, 171-178. (10.1002/evan.20185)

[18] Boesch C et al. 2020 Chimpanzee ethnography reveals unexpected cultural diversity. Nat. Hum. Behav. **4**, 910-916. (10.1038/s41562-020-0890-1) 32451479

[19] Luncz LV, Gill M, Proffitt T, Svensson MS, Kulik L, Malaivijitnond S. 2019 Group-specific archaeological signatures of stone tool use in wild macaques. eLife **8**, e46961. (10.7554/eLife.46961) 31635691 PMC6805154

[20] Lycett SJ, Collard M, McGrew WC. 2007 Phylogenetic analyses of behavior support existence of culture among wild chimpanzees. Proc. Natl Acad. Sci. USA **104**, 17 588-17 592. (10.1073/pnas.0707930104) PMC2077048 17968009

[21] Hill K et al. 2009 The question of animal culture. Cambridge, MA: Harvard University Press.

[22] Langergraber KE et al. 2011 Genetic and ‘cultural’ similarity in wild chimpanzees. Proc. R. Soc. B **278**, 408-416. (10.1098/rspb.2010.1112) PMC3013405 20719777

[23] Biro D, Haslam M, Rutz C. 2013 Tool use as adaptation. Phil. Trans. R. Soc. B **368**, 20120408. (10.1098/rstb.2012.0408) 24101619 PMC4027410

[24] Luncz LV, Sirianni G, Mundry R, Boesch C. 2018 Costly culture: differences in nut-cracking efficiency between wild chimpanzee groups. Anim. Behav. **137**, 63-73. (10.1016/j.anbehav.2017.12.017)

[25] Fragaszy DM, Biro D, Eshchar Y, Humle T, Izar P, Resende B, Visalberghi E. 2013 The fourth dimension of tool use: temporally enduring artefacts aid primates learning to use tools. Phil. Trans. R Soc. Lond. B **368**, 20120410. (10.1098/rstb.2012.0410) 24101621 PMC4027420

[26] Koops K, Visalberghi E, van Schaik CP. 2014 The ecology of primate material culture. Biol. Lett. **10**, 20140508. (10.1098/rsbl.2014.0508) 25392310 PMC4261853

[27] Visalberghi E, Spagnoletti N, da Silva ED R, Andrade FRD, Ottoni E, Izar P, Fragaszy D. 2009 Distribution of potential suitable hammers and transport of hammer tools and nuts by wild capuchin monkeys. Primates **50**, 95-104. (10.1007/s10329-008-0127-9) 19172379

[28] Claidière N, Messer EJE, Hoppitt W, Whiten A. 2013 Diffusion dynamics of socially learned foraging techniques in squirrel monkeys. Curr. Biol. **23**, 1251-1255. (10.1016/j.cub.2013.05.036) 23810529

[29] Barrett BJ, McElreath RL, Perry SE. 2017 Pay-off-biased social learning underlies the diffusion of novel extractive foraging traditions in a wild primate. Proc. R. Soc. B **284**, 20170358. (10.1098/rspb.2017.0358) PMC5474070 28592681

[30] Monteza-Moreno CM, Dogandžić T, McLean KA, Castillo-Caballero PL, Mijango-Ramos Z, Del Rosario-Vargas E, Crofoot MC, Barrett BJ. 2020 White-faced capuchin, *Cebus capucinus* imitator, hammerstone and anvil tool use in riparian habitats on Coiba Island, Panama. Int. J. Primatol. **41**, 429-433. (10.1007/s10764-020-00156-5)

[31] Lonsdorf EV. 2013 The role of mothers in the development of complex skills in chimpanzees. In Building babies: primate development in proximate and ultimate perspective (eds KBH Clancy, K Hinde, JN Rutherford), pp. 303-318. New York, NY: Springer.

[32] Tan AWY. 2017 From play to proficiency: the ontogeny of stone-tool use in coastal-foraging long-tailed macaques from a comparative perception-action perspective. J. Comp. Psychol. **131**, 89. (10.1037/com0000068) 28318292

[33] Lonsdorf EV. 2006 What is the role of mothers in the acquisition of termite-fishing behaviors in wild chimpanzees (*Pan troglodytes schweinfurthii*)? Anim. Cogn. **9**, 36-46. (10.1007/s10071-005-0002-7) 16195914

[34] Tan AWY, Hemelrijk CK, Malaivijitnond S, Gumert MD. 2018 Young macaques (*Macaca fascicularis*) preferentially bias attention towards closer, older, and better tool users. Anim. Cogn. **21**, 551-563. (10.1007/s10071-018-1188-9) 29754253

[35] Schuppli C, Forss SIF, Meulman EJM, Zweifel N, Lee KC, Rukmana E, Vogel ER, van Noordwijk MA, van Schaik CP. 2016 Development of foraging skills in two orangutan populations: needing to learn or needing to grow? Front. Zool. **13**, 43. (10.1186/s12983-016-0178-5) 27708679 PMC5041519

[36] Hopkins WD, Reamer L, Mareno MC, Schapiro SJ. 2015 Genetic basis in motor skill and hand preference for tool use in chimpanzees (*Pan troglodytes*). Proc. R. Soc. B **282**, 20141223. (10.1098/rspb.2014.1223) PMC4298198 25520351

[37] Hopkins WD, Latzman RD, Mareno MC, Schapiro SJ, Gómez-Robles A, Sherwood CC. 2019 Heritability of gray matter structural covariation and tool use skills in chimpanzees (*Pan troglodytes*): a source-based morphometry and quantitative genetic analysis. Cereb. Cortex **29**, 3702-3711. (10.1093/cercor/bhy250) 30307488 PMC6686745

[38] Krützen M, Mann J, Heithaus MR, Connor RC, Bejder L, Sherwin WB. 2005 Cultural transmission of tool use in bottlenose dolphins. Proc. Natl Acad. Sci. USA **102**, 8939-8943. (10.1073/pnas.0500232102) 15947077 PMC1157020

[39] Whiten A. 2000 Primate culture and social learning. Cogn. Sci. **24**, 477-508. (10.1207/s15516709cog2403_6)

[40] Vila-Pouca C, Vedder S, Kotrschal A. 2022 Hybridization may promote variation in cognitive phenotypes in experimental guppy hybrids. Am. Nat. **200**, 607-619. (10.1086/720731) 36150200

[41] Cortés-Ortiz L et al. 2017 Hybridization and hybrid zones. In The international encyclopedia of primatology (ed. M Bezanson etal), pp. 1-5. Hoboken, NJ: John Wiley & Sons.

[42] Cortés-Ortiz L, Agostini I, Aguiar LM, Kelaita M, Silva FE, Bicca-Marques JC. 2015 Hybridization in howler monkeys: current understanding and future directions. In Howler monkeys: adaptive radiation, systematics, and morphology (eds MM Kowalewski, PA Garber, L Cortés-Ortiz, B Urbani, D Youlatos), pp. 107-131. New York, NY: Springer.

[43] Tan A, Tan SH, Vyas D, Malaivijitnond S, Gumert MD. 2015 There is more than one way to crack an oyster: identifying variation in burmese long-tailed macaque (*Macaca fascicularis aurea*) stone-tool use. PLoS ONE **10**, e0124733. (10.1371/journal.pone.0124733) 25970286 PMC4430286

[44] Luncz LV, Svensson MS, Haslam M, Malaivijitnond S, Proffitt T, Gumert M. 2017 Technological response of wild macaques (*Macaca fascicularis*) to anthropogenic change. Int. J. Primatol. **38**, 872-880. (10.1007/s10764-017-9985-6) 29056799 PMC5629225

[45] Gumert MD, Malaivijitnond S. 2012 Marine prey processed with stone tools by burmese long-tailed macaques (*Macaca fascicularis aurea*) in intertidal habitats. Am. J. Phys. Anthropol. **149**, 447-457. (10.1002/ajpa.22143) 23042618

[46] Bandini E, Tennie C. 2018 Naive, captive long-tailed macaques (*Macaca fascicularis fascicularis*) fail to individually and socially learn pound-hammering, a tool-use behaviour. R. Soc. Open Sci. **5**, 171826. (10.1098/rsos.171826) 29892375 PMC5990768

[47] Krause J, Lusseau D, James R. 2009 Animal social networks: an introduction. Behav. Ecol. Sociobiol. **63**, 967-973. (10.1007/s00265-009-0747-0)

[48] Haythornthwaite C, de Laat M. 2010 Social networks and learning networks: using social network perspectives to understand social learning. Proc. 7th Int. Conf. Networked Learning **2**, 183-190.

[49] Farine DR, Whitehead H. 2015 Constructing, conducting and interpreting animal social network analysis. J. Anim. Ecol. **84**, 1144-1163. (10.1111/1365-2656.12418) 26172345 PMC4973823

[50] Franz M, Nunn CL. 2009 Network-based diffusion analysis: a new method for detecting social learning. Proc. R. Soc. B **276**, 1829-1836. (10.1098/rspb.2008.1824) PMC2674490 19324789

[51] Hoppitt W, Boogert NJ, Laland KN. 2010 Detecting social transmission in networks. J. Theor. Biol. **263**, 544-555. (10.1016/j.jtbi.2010.01.004) 20064530

[52] Allen J, Weinrich M, Hoppitt W, Rendell L. 2013 Network-based diffusion analysis reveals cultural transmission of lobtail feeding in humpback whales. Science **340**, 485-488. (10.1126/science.1231976) 23620054

[53] Alberts SC, Altmann J. 2012 The amboseli baboon research project: 40 years of continuity and change. In Long-Term field studies of primates (eds PM Kappeler, DP Watts), pp. 261-287. Berlin, Heidelberg: Berlin, Germany: Springer.

[54] Fooden J. 1995 Systematics review of Southeast Asian longtail macaques, *Macaca fascicularis* (Raffles, 1821). Fieldiana: Zool., ns **81**, 2-3.

[55] Phadphon P, Kanthaswamy S, Oldt RF, Hamada Y, Malaivijitnond S. 2022 Population structure of *Macaca fascicularis aurea*, and their genetic relationships with *M. f. fascicularis* and *M. mulatta* determined by 868 RADseq-derived autosomal SNPs—a consideration for biomedical research. J. Med. Primatol. **51**, 33-44. (10.1111/jmp.12554) 34825374 PMC8849537

[56] Csardi G, Nepusz T. 2006 The igraph software package for complex network research. Inter J. Complex Syst. **1695**, 1-9.

[57] R Core Team. 2021 R: a language and environment for statistical computing. Vienna, Austria: R Foundation for Statistical Computing.

[58] Kulahci IG, Ghazanfar AA, Rubenstein DI. 2018 Knowledgeable lemurs become more central in social networks. Curr. Biol. **28**, 1306-1310.e2. (10.1016/j.cub.2018.02.079) 29628372

[59] Acerbi A, Smolla M, Mesoudi A. 2020 Individual-based models of cultural evolution. London, UK: Routledge.

[60] Masad D, Kazil J. 2015 MESA: an agent-based modeling framework. In Proc. of the 14th Python in Sci. Conf. (SCIPY 2015), pp. 53-60. See https://conference.scipy.org/proceedings/scipy2015/jacqueline_kazil.html.

[61] Rossum GV. 1995 Python tutorial, technical report CS-R9526. Amsterdam, The Netherlands: Centrum voor Wiskunde en Informatica.

[62] Grimm V, Berger U, DeAngelis DL, Polhill JG, Giske J, Railsback SF. 2010 The ODD protocol: a review and first update. Ecol. Modell **221**, 2760-2768. (10.1016/j.ecolmodel.2010.08.019)

[63] Stan Development Team. 2022 RStan: the R interface to Stan. R package version 2.522. See https://mcstan.org/rstan/articles/rstan.html.

[64] McElreath R. 2020 Statistical rethinking: a Bayesian course with examples in R and Stan. London, UK: Chapman and Hall.

[65] Laland K, Janik V. 2006 The animal cultures debate. Trends Ecol. Evol. **21**, 542-547. (10.1016/j.tree.2006.06.005) 16806574

[66] Matsunaga E, Nambu S, Oka M, Tanaka M, Taoka M, Iriki A. 2015 Identification of tool use acquisition-associated genes in the primate neocortex. Dev. Growth Differ. **57**, 484-495. (10.1111/dgd.12227) 26173833 PMC11520950

[67] Whiten A, Ayala FJ, Feldman MW, Laland KN. 2017 The extension of biology through culture. Proc. Natl Acad. Sci. USA **114**, 7775-7781. (10.1073/pnas.1707630114) 28739924 PMC5544333

[68] Boyd R, Richerson PJ, Henrich J. 2011 The cultural niche: why social learning is essential for human adaptation. Proc. Natl Acad. Sci. USA **108**, 10 918-10 925. (10.1073/pnas.1100290108) PMC3131818 21690340

[69] Sanz CM, Morgan DB. 2013 Ecological and social correlates of chimpanzee tool use. Phil. Trans. R. Soc. B **368**, 20120416. (10.1098/rstb.2012.0416) 24101626 PMC4027411

[70] Boesch C. 2014 Wild cultures a comparison between chimpanzee and human cultures. Cambridge, UK: Cambridge University Press.

[71] Reeves JS, Tan A, Malaivijitnond S, Luncz LV. 2023 Data from: Simulation and social network analysis provide insight into the acquisition of tool behaviour in hybrid macaques. Dryad Digital Repository. (10.5061/dryad.d2547d86b). PMC10050919 36987645

[72] Reeves JS, Tan A, Malaivijitnond S, Luncz LV. 2023 Simulation and social network analysis provide insight into the acquisition of tool behaviour in hybrid macaques. Figshare. (10.6084/m9.figshare.c.6471274) PMC10050919 36987645

